# Basal Cell Carcinoma with Sarcomatoid Differentiation—A Rare Type and Its Possible Origin

**DOI:** 10.3390/dermatopathology12030020

**Published:** 2025-07-08

**Authors:** Nessr Abu Rached, Natalie Orlinski, Eggert Stockfleth, Markus Stücker, Martin Doerler

**Affiliations:** Skin Cancer Center, Department of Dermatology, Venereology and Allergology, Ruhr-University Bochum, 44791 Bochum, Germany; natalie.orlinski@rub.de (N.O.); eggert.stockfleth@klinikum-bochum.de (E.S.); markus.stuecker@kklbo.de (M.S.); martin.doeler@kklbo.de (M.D.)

**Keywords:** sarcomatoid, basal cell carcinoma, dermatopathology, basal cell carcinoma with sarcomatoid differentiation

## Abstract

Background: We present an interesting case involving a tumour comprising both basal cell tumour cells and sarcomatoid tumour cells. An 86-year-old woman presented with an erythematous lesion on her left cheek. Clinical and dermoscopic findings suggested BCC. Complete excision and histopathological examination revealed a BCC with a separate proliferation of atypical spindle and epithelioid cells. Immunohistochemical staining supported the diagnosis, with basaloid cells positive for CK5/6 and Ber-EP4 and sarcomatoid cells positive for CD10 and vimentin. Results: Histology and immunohistochemistry confirmed a basal cell carcinoma with sarcomatoid differentiation. The close proximity of sarcomatoid cells to the BCC component suggests a potential role of epithelial–mesenchymal interactions in tumour development. Further investigations into the exact origin of this tumour are required. Conclusion: Basal cell carcinoma with sarcomatoid differentiation is rare. This case highlights the importance of thorough histological and immunohistochemical evaluation. Further studies are necessary to better understand the pathogenesis of such collision tumours.

## 1. Introduction

We present a rare histopathological case of a tumour consisting of a basal cell carcinoma (BCC) and sarcomatoid tumour cells. Basal cell carcinoma histologically shows a proliferation of basaloid cells and embryologically originates from the ectoderm [1,2,3]. To date, only a few cases of basal cell carcinoma with sarcomatoid differentiation have been reported.

## 2. Histopathology Case

**Figure 1:**
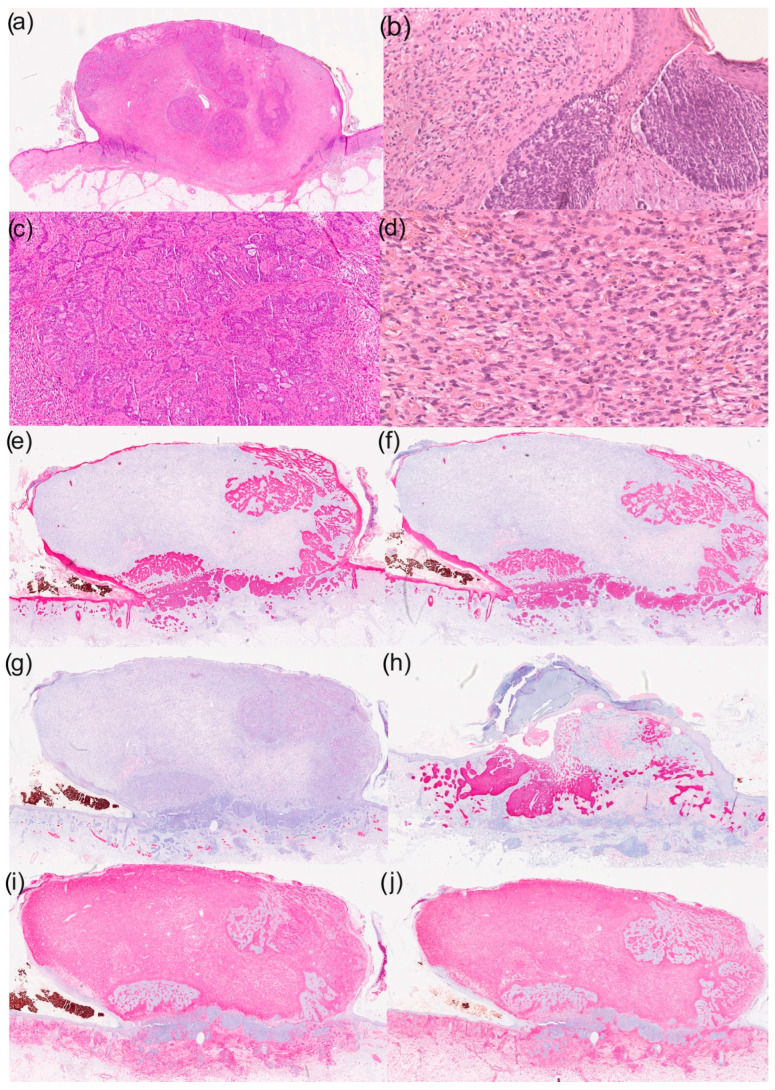
Histological and immunohistochemical results of a basal cell carcinoma with sarcomatoid differentiation: (**a**) overview (haematoxylin–eosin staining; HE staining), (**b**) parts of the basal cell carcinoma with typical morphological characteristics (100× magnification, HE staining), (**c**) reticular growth pattern of basal cell carcinoma (50× magnification with HE staining), (**d**) cell- and vessel-rich stroma with epithelioid tumour cells (200× magnification with HE staining), (**e**) immunohistochemistry for CK5/6 (overview), (**f**) immunohistochemistry for CK MNF (overview), (**g**) immunohistochemistry for desmin (overview), (**h**) immunohistochemistry for Ber-EP4 (overview), (**i**) immunohistochemistry for CD10 (overview) and (**j**) immunohistochemistry for vimentin (overview).

**Figure 2:**
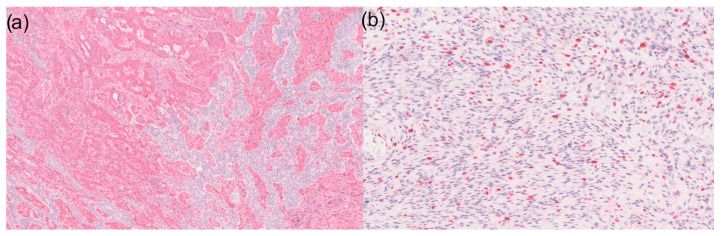
(**a**) 50× magnification of a basal cell carcinoma with sarcomatoid differentiation in immunohistochemistry for vimentin: Sarcomatoid tumour components are visible on the left and basal cell carcinoma on the right side. However, it is noticeable that CD10-positive tumour cells can also be found within the basal cell carcinoma; (**b**) Ki67 expression shows high expression in the sarcomatoid tumour area (100× magnification).

An 86-year-old woman presented to our dermatology department with a skin lesion on her left cheek that was clinically suspected to be BCC. According to her medical history, the lesion had progressed in the past few months. Clinical examination revealed an erythematous lesion approximately 1 cm in diameter. Dermoscopic examination of the lesion showed telangiectasia and a central scar. A complete excision and a histological analysis were performed. We found histopathological characteristics of conventional BCC (basaloid nests with peripheral palisading and cleft formation) (Figure 1 and Figure 2). In addition, other tumour components were found next to and between the basaloid cells. This second tumour component showed epithelioid and spindled cell proliferations (Figure 2). Immunohistochemical analyses were performed to further characterise this tumour. Immunohistochemically, basaloid tumour cells were CK5/6- and Ber-EP4-positive and CK20-negative. The epithelioid tumour cells were strongly CD10- and vimentin-positive and actin-, desmin-, CD34- and S100-negative. After histological and immunohistochemical correlation, the diagnosis of a basal cell carcinoma with sarcomatoid differentiation was made.

## 3. Discussion and Conclusions

BCCs with sarcomatoid differentiation are a rare and heterogeneous tumour entity that has only been described in a few case reports and small case series to date. The studies published to date (Table 1) consistently show a biphasic architecture consisting of an epithelial BCC component and a sarcomatoid, spindle cell or heterologously differentiated mesenchymal component. Histologically, the mesenchymal components vary from undifferentiated spindle cell proliferations to osteosarcoma-like differentiation [2] to atypical fibroxanthoma-like patterns [4]. Immunohistochemically, the mesenchymal components sometimes show coexpression of epithelial markers (e.g., pancytokeratins, EMA) and mesenchymal markers (e.g., vimentin, smooth muscle actin), which supports the idea of translinear or dedifferentiating development. In individual cases, molecular abnormalities such as a PTCH1 mutation [5] or an EWSR1-PBX1 gene fusion [3] have been detected.

In the literature, a distinction is made between a collision tumour and a tumour with biphasic components. A collision tumour is defined as the coexistence of two or more histologically distinct tumours at the same anatomic site with clearly demarcated borders [6]. Our case most likely represents a biphasic, sarcomatoid BCC, which is a tumour composed of a malignant epithelial component intimately associated with a malignant mesenchymal component.

Basal cell carcinoma is the most common malignant skin tumour and originates from the basal cell layer of the epidermis. Embryologically, the basal cell layer and the epidermis develop from the ectoderm [7]. Interestingly, in the area of the stroma of the BCC, CD10- and vimentin-positive sarcomatoid tumour cells were found in our case. CD10- and vimentin-positive tumour cells were also found between the reticular basaloid cell proliferates (Figure 2). The expression of CD10 in the stroma of BCCs varies depending on the histological subtype [8]. Whether the sarcomatoid component originates from the stromal mesenchymal cells of a BCC or is the result of metaplastic transformation of the carcinomatous component remains speculative [1,2,3,9]. Furthermore, the origin of this rare tumour could be explained by the embryogenesis of the skin. During embryogenesis, the dermis develops primarily from the mesoderm [10]. Sarcomatoid tumour cells are dermal, suggesting a mesenchymal origin. However, the ectodermal (epithelial) component is also known to be able to induce the mesoderm [10]. These epithelial–mesenchymal interactions influence the extracellular matrix and microarchitecture at the dermal–epidermal junction. This interaction may be the origin of BCC with sarcomatoid differentiation. This hypothesis cannot be proven based on our observations, but further research should be conducted to support or refute this hypothesis. Examination of the cases suggests that BCC with sarcomatoid differentiation arises from a clonal epithelial precursor that undergoes dedifferentiation. This hypothesis is supported by immunohistochemical evidence of identical marker expression (e.g., p53) in both components [11]. Several case reports have histologically demonstrated the transition from epithelial to mesenchymal components [2,12], which could indicate epithelial–mesenchymal transition. The detection of a gene fusion [3] raises the question of whether true myoepithelial differentiation is present in individual cases.

Basal cell carcinoma with sarcomatoid differentiation is a potentially aggressive tumour [11,13]. From a therapeutic perspective, complete surgical excision appears to remain the central approach. The published cases with sufficient follow-up show that no recurrences or metastases occurred following the removal of the entire tumour, even in cases of heterologous differentiation. However, the number of cases is small and further follow-up is required to definitively evaluate the actual recurrence and metastasis potential of these rare tumours.

In conclusion, a basal cell carcinoma with sarcomatoid differentiation is rare and its origin should be investigated further.

**Table 1:** A brief overview of BCC with sarcomatoid differentiation in the literature.

Author (Year)	Article Type, Number of Cases	Histology	Conclusion
Menamin et al. (2016) [2]	Case series, n = 11	- All tumours were located in the dermis; some with subcutaneous infiltration. - In 10 cases, in addition to the BCC component, there was a heterologous, osteosarcomatous and undifferentiated sarcoma. - Mesenchymal components showed partial positivity for epithelial markers (e.g., pancytokeratins, EMA) and smooth muscle actin, which supports the transition between cell types.	- Rare subtype of BCC. - After complete surgical removal, the outcome was favourable in all cases.
Mestre-Alagarda et al. (2019) [3]	Case report, n = 1	- A small, conventional nodular BCC component that transitioned into a predominantly spindle cell-containing, epithelial-like growth. - The BCC was BER-EP4-positive. - The sarcomatoid component was positive for α-SMA, MSA, calponin and p63 - Partial positive for S100 and GFAP in epithelial-like cells.	- Detection of EWSR1-PBX1 gene fusion, indicating true myoepithelial carcinoma differentiation. - Biphasic sarcomatoid BCC with myoepithelial carcinoma differentiation.
Kiuru et al. (2014) [5]	Case report, n = 1	- Biphasic architecture with a basaloid epithelial component showing typical features of basal cell carcinoma (basaloid cells with palisade-like arrangement) and a sarcomatoid, spindle-cell component.	- Basal cell carcinosarcoma with PTCH1 mutations in both epithelial and sarcomatoid primary tumour components.
Rose et al. (2008) [12]	Case series, n = 5	- A typical nodular basal cell carcinoma with a stromal component consisting of atypical spindle cells and tumour giant cells, undifferentiated stroma or osteoid formation.	- None showed signs of recurrence following complete resection.
Bigby et al. (2005) [11]	Case report, n = 1	- Biphasic structure with an epithelial component exhibiting features of BCC and a sarcomatoid component with pleomorphic spindle cells	- Carcinosarcomas of the skin probably arose from dedifferentiation of an epithelial tumour, which is supported by p53 expression in both components.
Inaloz et al. (2003) [4]	Case report, n = 1	- An exophytic skin tumour with pleomorphic spindle cells consistent with atypical fibroxanthoma and a basaloid epithelial component showing typical BCC features.	- A carcinosarcomatous skin tumour with biphasic architecture could have originated from undifferentiated progenitor cells.

BCC, basal cell carcinoma.

## Data Availability

Data is contained within the article.
